# Prevalence and Risk Factors Predicting Onychomycosis in Patients with and Without Diabetes Mellitus in Spain: A Cross-Sectional Study

**DOI:** 10.3390/jof10110790

**Published:** 2024-11-14

**Authors:** David Navarro-Pérez, José Luis Lázaro-Martínez, Sara García-Oreja, Teresa Pérez-Pérez, Francisco Javier Álvaro-Afonso, Aroa Tardáguila-García

**Affiliations:** 1 Diabetic Foot Unit, University Podiatric Clinic, Faculty of Nursing, Physiotherapy and Podiatry, Complutense University of Madrid, 28040 Madrid, Spain; davinava@ucm.es (D.N.-P.); sagarc14@ucm.es (S.G.-O.); alvaro@ucm.es (F.J.Á.-A.); aroa.tardaguila@ucm.es (A.T.-G.); 2 San Carlos Clinical Hospital Health Research Institute (IdISSC), 28040 Madrid, Spain; 3 Department of Statistics and Data Science, Complutense University of Madrid, 28040 Madrid, Spain; teperez@ucm.es; 4 Institute of Statistics and Data Science, Complutense University of Madrid, 28040 Madrid, Spain

**Keywords:** onychomycosis, diabetes mellitus, diabetic foot, risk factors, diagnosis, prevalence

## Abstract

Background: A cross-sectional study was conducted to investigate the prevalence of onychomycosis (ONM) and its causative pathogens in populations with and without diabetes in Spain. The association between the presence of ONM, different risk factors, and comorbidities was also examined. Methodology: A total of 160 patients with diabetes and 160 individuals without diabetes were recruited consecutively. A single investigator recorded the relative data of each patient and sampled nail dust and detritus for microbiological culture and polymerase chain reaction (PCR) analyses of patients who showed clinical signs of fungal infection. Results: The prevalence of ONM was 36.88% (59/160) in the population with diabetes, 17.5% (28/160) in the population without diabetes, and 34.35% (45/131) in the population with diabetic foot. Dermatophyte fungi were most frequently identified, although the proportion was higher among those without diabetes than those with diabetes (19/28 and 28/59, respectively). However, the rate of mixed infections was higher in the population with diabetes compared to those without diabetes (13/59 and 2/28, respectively). A statistically significant association was found between the presence of diabetes and the risk of ONM (*p* < 0.001; odds ratio (OR) = 2.754; 95% confidence interval (CI) 1.652–4.679). The risk factors associated with ONM among the patients with diabetes were a history of minor amputation, revascularisation, or cardiovascular disease, a low educational level, HbA1c values > 7%, hyperkeratosis, and subungual detritus. Among the patients without diabetes, nail thickening and chromonychia were associated with ONM. Conclusion: The results of this study suggest that the early diagnosis of ONM and knowledge of risk factors among patients with diabetes could enable the prevention of ONM, complications, and serious injuries through education for professionals and patients.

## 1. Introduction

The occurrence of diabetes mellitus (DM) continues to increase worldwide, with the world’s population of people with diabetes increasing from around 422 million in 2019 to 537 million in 2024 [1,2]. Currently, Spain has the second-highest prevalence of DM among the countries in Europe with a range of 10.3%, when adjusted for age (20–79 years), to 14.8% [3]. DM is associated with multiple complications, such as neuropathy, retinopathy, nephropathy, peripheral vascular disease (PVD), cardiovascular disease, and diabetic foot disease. Diabetic foot disease in particular is the leading cause of non-traumatic amputations, is preceded by ulcers, and causes morbidity, disability, and mortality [4,5,6,7].

In cases of normal blood glucose, insulin modulates neutrophil function in healthy adults and acts as an immunoregulator of immune cells [8,9,10]. Long-term hyperglycaemia weakens the immune system and white blood cells, which leads to an increase in bacterial and fungal infections [11,12,13]. Therefore, under these conditions, fungi and opportunistic pathogens contribute to the development of fungal and bacterial infections, and the risk of developing onychomycosis (ONM) is almost three times higher than the risk among people without diabetes [7,11,14,15]. The overall prevalence of ONM among people with diabetes is about one-third [15,16,17].

ONM is a fungal infection that can cause the thickening of the nails and subungual hyperkeratosis, which can lead to surrounding lesions and ulcers that can act as an entry point for other microorganisms [18,19]. In fact, in previous studies, ONM has also been associated with the development of diabetic foot ulcers (odds ratio (OR) = 1.58; 95% confidence interval (CI): 1.16–2.16) [20,21,22]. In general, mild ONM is not a major problem, but more severe ONM can be an issue for those who are not diagnosed and treated promptly and for patients with neuropathy and PVD. One of the reasons for this is that thickened nails can increase pressure on the nail plate and injure the subungual and surrounding skin [23,24]. Neuropathic patients do not feel these small lesions, which become an entry point for bacteria and can cause more severe infections that endanger the affected toes and even the limbs, depending on the severity and extent. Therefore, special care must be taken for subungual lesions due to the high risk of osteomyelitis resulting from the short distance between the nail bed and the bone. In combination with impaired circulation among patients with PVD, these conditions can delay ulcer healing and increase the risk of amputation or even lead to death [7,19,23,24,25,26,27,28,29,30]. The prevention, early diagnosis, and treatment of ONM while it is still mild are increasingly important due to the increasing prevalence of DM and the difficult therapeutic management of ONM [2,22,29,31,32,33,34].

Aragón-Sánchez et al. [14] conducted a retrospective study in Spain and included 101 patients with DM and signs of ONM. They found that 40.6% of the patients had ONM after diagnosis by laboratory tests, and *Trichophyton rubrum* was the most frequently detected pathogen. However, only patients with a clinical suspicion of ONM were included, and patients with clinically healthy nails were excluded, so the observed prevalence was not fully representative of all patients with diabetes.

The prevalence of ONM among patients with diabetes has been estimated in countries such as Italy, Germany, Turkey, and India, among others, but no other study has examined the prevalence of and risk factors associated with ONM in a Spanish population with diabetes, whether or not they have clinical signs of ONM infection. Therefore, the aim of this study was to estimate and compare the prevalence of ONM among patients with and without diabetes. Furthermore, the factors associated with the occurrence of ONM were analysed with adjustment for the presence or absence of diabetes, and possible confounding factors were identified. Lastly, patients with and without diabetes were compared in terms of the risk factors for ONM.

## 2. Materials and Methods

### 2.1. Participants and Samples

This study was conducted in accordance with the Declaration of Helsinki and current national legislation governing research involving patients [35]. Prior to participation in the study, the patients signed informed consent forms, and the study has been approved by an ethics committee (code 23/610-E). A cross-sectional cohort study was conducted between October 2023 and April 2024 with 320 patients (160 patients with DM and 160 without DM) who were treated at a specialised foot clinic in Madrid, Spain. Patients were recruited sequentially.

The inclusion criteria were an age greater than 18 years, DM type 1 (DM1) or type 2 (DM2) with or without clinical signs of ONM infection, and patients without diabetes with or without clinical signs of ONM infection. Patients were excluded if they were previously diagnosed with ONM, had psoriasis or subungual tumours, or were receiving immunosuppressive therapy. Only patients with clinical signs of infection on dermatoscopic inspection (nail thickening, subungual hyperkeratosis, chromonychia, onycholysis, dermatophytoma, subungual detritus, brittle nail, or longitudinal striae) were sampled for culture and polymerase chain reaction (PCR) analyses [36,37]. The sampling was carried out by the same experienced investigator by cutting the nails, taking scrapings of subungual hyperkeratosis or detritus, and milling the nail plate. The sample selection required sufficiently large portions of the nail to allow for both diagnostic analyses.

The samples were examined by microbiological culture on Sabourad dextrose agar for 1 to 3 weeks. PCR was performed based on the protocols of previous studies [37,38]. After 1 to 3 weeks, the laboratory provided the results. The PCR results indicated whether samples were positive or negative only for dermatophytes, and the microbiological culture indicated a “positive” or “negative” status for the dermatophytes and non-dermatophyte moulds. The nail plates of all sampled patients were photographed, and then the ONM severity index (OSI) was calculated for patients who tested positive [39]. The patients were then asked a series of questions about their current health status, irrespective of the culture and PCR results.

### 2.2. Statistical Analysis

Samples were described using tables. Qualitative variables were summarised using frequencies and percentages, and quantitative variables were summarised using their means and standard deviations or their medians and interquartile ranges as appropriate. The prevalence of ONM in the two groups was estimated along with the corresponding 95% CIs. Logistic regression models were adjusted to determine whether there was an association between nail infection, diabetes, and comorbidities. First, univariate models were constructed, and variables with *p*-values < 0.2 were selected for inclusion in the multivariate model.

A subgroup analysis was carried out using logistic regression models to analyse risk factors for ONM among patients with and without diabetes. As this study was an observational study where the patients were not randomised, differences between those with and without diabetes may have biassed the estimates. To analyse the possible impact of non-measurable confounding factors on the association between diabetes and nail infection, a sensitivity analysis was carried out by including the propensity score in the logistic regression model [40] to balance the characteristics of both groups.

To estimate the propensity score, the logistic regression model was first adjusted. Diabetes was the dependent variable while the independent variables were characteristics associated with diabetes that also constitute risk factors for nail infection. Specifically, the model included age, sex, body mass index (BMI), smoking status (yes/no), and physical activity (sedentary/active). Statistical analyses were carried out using the statistical software IBM SPSS version 25.0 (SPSS Inc., Chicago, IL, USA). In all analyses, *p* < 0.05 was considered statistically significant.

### 2.3. Sample Size

To achieve the main objective, sample size estimation was performed using the sample size calculator GRANMO version 7.12 (Institut Municipal d’Investigació Médica, Barcelona, Spain). The global prevalence of ONM is 5.5% [41], and the risk of ONM is three times higher among patients with diabetes [15,41,42]. The calculation was conducted using these figures along with an alpha risk of 0.05, a beta risk of 0.2, a bilateral contrast, and a loss to follow-up rate of 10%. The results indicated that 160 participants in the group with DM and 160 in the group without DM would be required.

## 3. Results

### 3.1. Study Population

A total of 320 patients were included, which comprised 160 patients with diabetes (23 with DM1 and 137 with DM2) and 160 patients without diabetes. There were 184 males (128 with diabetes and 56 without diabetes) and 136 females (32 with diabetes and 104 without diabetes). The mean age was 68.41 ± 10.85 years for those with diabetes and 65.44 ± 12.67 years for those without diabetes.

A total of 73.8% (118/160) of the population with diabetes had a history of ulceration, and 28.1% (45/160) had minor amputations, while in the population without diabetes, 5% had ulceration (8/160), and 1.9% had minor amputations (3/160). In addition, 28.1% (45/160) of the population with diabetes and 2.5% (4/160) of the population without diabetes currently had active ulcers. In the population with diabetes, 65.6% (105/160) had neuropathy, 48.8% (78/160) had peripheral vascular disease, and 81.9% (131/160) had diabetic foot syndrome. Furthermore, 40% (64/160) had glycosylated haemoglobin values above 7%, and 55% (88/160) had baseline blood glucose values above 126 mg/dL. Other data about the population can be seen in Table 1 and Table 2.

### 3.2. Prevalence and Clinical Signs of ONM

Dermatoscopy indicated clinical signs of ONM in 125 patients (86 with diabetes and 39 without diabetes). After combining the results of the microbiological culture and PCR, an ONM prevalence of 36.88% (59/160) was observed in the population with diabetes, and the rate was higher among those with DM2 (38.7%; 53/137) than those with DM1 (26.1%; 6/23). The prevalence in the population without diabetes was 17.5% (28/160). Of the 160 patients with diabetes, 131 had diabetic foot, of which 45 had ONM (34.35%).

The most frequently found clinical signs were chromonychia (73.3% with diabetes and 76.9% without diabetes), subungual hyperkeratosis (72.1% with diabetes and 66.7% without diabetes), and nail thickening (70.9% with diabetes and 74.4% without diabetes). Of the 160 patients with diabetes, 86 (53.75%) had nail changes, of which 59 (68.60%) had ONM (*p* < 0.001). Furthermore, among all 320 patients, 5 had subungual ulceration, and all 5 of them had DM and ONM (*p* = 0.003). Table 3 shows the remaining data on the prevalence and clinical signs.

### 3.3. Pathogens Detected and Severity of ONM

Of the 87 cases with ONM, 47 involved dermatophytes, 15 involved mixed infections, 14 involved yeasts, and 11 involved non-dermatophyte filamentous fungi. For both patients with and without diabetes, dermatophyte fungi were most frequently identified, although the proportion was higher in the group without diabetes (28/59 and 19/28, respectively). However, the proportion of mixed infections was higher in the population with diabetes compared to those without diabetes (13/59 and 2/28, respectively).

In the microbiological culture, the most frequently found microorganisms were mixed infections (15/54), *Candida* sp. (14/54), and *T. rubrum* (11/54). Among those with and without diabetes, the most frequents types of ONM were total dystrophic ONM (52.5% and 64.3%, respectively) and severe ONM (81.4% and 82.1%, respectively). The remaining data on the ONM can be found in Table 3.

### 3.4. Associated Risk Factors

The logistic regression model showed a statistically significant association between the presence of diabetes and the risk of ONM (*p* < 0.001; OR = 2.754; 95% CI 1.652–4.679). According to the subgroup analysis, in the group with diabetes, the presence of ONM was statistically significantly associated with a history of minor amputations (*p* = 0.014; OR = 4.493; 95% CI 1.356–14.881), a history of revascularisation (*p* = 0.04; OR = 5.879; 95% CI 1.083–31.922), a history of cardiovascular disease (*p* < 0.001; OR = 10.046; 95% CI 3.322–30.381), a low educational level (*p* = 0.006, OR = 1.478; 95% CI 1.210–4.796), and HbA1c values greater than 7% (*p* = 0.008; OR = 4.036; 95% CI 1.448–11.253). Clinical signs associated with the presence of ONM were subungual hyperkeratosis (*p* = 0.001; OR = 6.573; 95% CI 2.060–20.976) and subungual detritus (*p* = 0.032; OR = 3.660; 95% CI 1.117–11.995).

In the group without diabetes, the presence of ONM was significantly associated with only nail thickening (*p* = 0.01; OR = 12.135; 95% CI 1.806–81.518) and chromonychia (*p* = 0.026; OR = 9.232; 95% CI 1.303–65.388). In the whole study population, a statistically significant association was also found between a higher number of medications taken (polymedicated patients, more than five medications [43]) and the presence of ONM (*p* = 0.008; OR = 1.956; 95% CI 1.188–3.218). The presence of diabetes was also statistically associated with the number of medications taken (*p* < 0.001; OR = 2.015; 95% CI 1.732–2.344).

The presence of diabetes was not statistically significantly associated with the type of fungus (*p* = 0.128), OSI (*p* = 0.829), type of ONM (*p* = 0.09), or affected area (*p* = 0.456), but it did have an association with the affected nail quarter (*p* = 0.026). The type of fungus was not associated with a higher OSI (*p* = 0.621) and HbA1c > 7% (*p* = 0.752). The remaining values are shown in Table 4.

### 3.5. Sensitivity Analysis of Association Between DM and ONM

When the propensity score was included in the logistic regression model, the results obtained were very similar (*p* < 0.001; OR = 2.351; 95% CI 1.295–4.356). Therefore, although no causal effect could be determined, the data suggest that diabetes has a significant impact on the presence of ONM. Furthermore, the results imply that individuals with diabetes have almost three times more of a risk of developing ONM than those without diabetes.

## 4. Discussion

The complications of diabetes associated with ONM have not been extensively studied. The current study indicated a prevalence of ONM of 36.88% in Spanish patients with diabetes (59/160), and the rate was higher among those with DM2 (38.7%; 53/137) than those with DM1 (26.1%; 6/23). These data are similar to those of a study conducted in Germany [33], which showed an ONM prevalence of 30.53% in a population with diabetes. The results are also similar to findings from India (17–34%) [29,44,45], Mexico (28% for those with DM2) [46], Turkey (12.8–37.8% for those with DM2) [47,48], Thailand (30.56%) [49], and Taiwan (30.76%) [50]. However, the present results are higher than rates observed in Kuwait (18.6%) [51] and Denmark (22%) [52], while they are lower than rates in Cameroon (50.7%) [19], Italy (53.3%) [32,53], and Malaysia (81.5%) [54]. In addition, 76.27% (45/59) of patients with diabetes and ONM had diabetic foot.

Statistically significant results were found between the presence of diabetes and the risk of ONM, and the prevalence of ONM was higher among patients with diabetes (36.88%; 59/160) than those without diabetes (17.5%; 28/160) (*p* < 0.001; OR = 2.754; 95% CI 1.652–4.679). The OR was similar to that reported by Gupta et al. [15,42]. However, the prevalence in the population without diabetes was higher than the rate of 5.5% reported by Gupta et al. [41]. This may be due to the fact that the included patients without diabetes regularly visited podiatrists and dermatologists, and ONM accounted for 23% of the reasons for consultation [55]. Therefore, we believe that the prevalence was probably higher because only patients who visited a podiatrist were included.

The most frequently isolated pathogens in the population with diabetes were dermatophytes (47.5%; 28/59) among patients with diabetic foot (42.5%; 19/45) and without diabetic foot (64.3%; 9/14). This is similar to previous findings [13,16,18,19,32,46,51,53]. Other studies suggest that non-dermatophyte fungi [29,44,45,54,56,57] and yeasts [29,47,48,58] are more common among patients with diabetes. However, in the present study, the prevalence of mixed infections was higher among the patients with diabetes (22%; 13/59) than those without diabetes (7.1%; 2/28). It was also more prevalent among the patients with diabetic foot (26.7%; 12/45) than those without diabetic foot (7.1%; 1/14). The fact that mixed infections are more prevalent in populations with diabetes may be due to their weak immune systems, promoting infection by another pathogen when conditions are optimal, combining dermatophyte and non-dermatophyte infections, and making the management of onychomycosis more difficult [11,12,13,14,15]. These differences could be due to differences in climatic, socio-economic, and cultural factors in different countries. Therefore, clinical diagnosis without the proper identification of microorganisms may lead to treatment failure [13,36].

In previous studies, the most frequent clinical presentation was OMDL [19,29,44,45,46,51], but in the present study, it was total dystrophic ONM (52.5%; 31/59). This could be due to a higher prevalence of non-dermatophyte fungal infections, the presence of PVD [7,11], and the high HbA1c levels of the patients included in the study, which may have led to nail thickening and subungual hyperkeratosis [45,50]. In fact, of the 86 patients with diabetes who had nail abnormalities, 59 were diagnosed with ONM. Furthermore, as in previous studies [15,46,54,56], the presence of abnormal-looking nails was significantly associated with the presence of ONM (*p* < 0.001; OR = 2.289; 95% CI 2.137–4.466), especially among those with all four nail quarters affected.

The multivariate analysis indicated that the statistically significant predictors of ONM were a history of minor amputations, a history of revascularisation, a history of cardiovascular disease, a low education level, and high HbA1c levels. The confounding factors identified were a history of ulcers, PVD, living in a rural area, and high baseline blood glucose values. However, all patients with minor amputations had a history of ulceration, and all revascularised patients had previous PVD. Therefore, although no statistically significant association was found between diabetic foot and the presence of ONM, vigilance is needed for this particular population as all the patients with minor amputations had diabetic foot. Lesions or ulcers caused by ONM could cause serious problems in such patients [59].

In contrast, no association was observed between ONM and other risk factors that have been described in other studies, such as the duration of diabetes [29,45,47,48,56], advanced age [29,33,45,46,48,49,51,54,56], male sex [29,33,45,49], diabetic neuropathy and neuroischaemic foot [14,29,47,51], DM2 [56], retinopathy [29,47,51], closed shoes [45], agriculture-related professions [49] or a rural area of residence, high triglyceride levels [48,50], obesity [50], a weak immune system [51], poor foot hygiene [45], increased nail plate thickness [50], low socio-economic status, and PVD [16,29,48,54,60]. In other studies, no association was observed with sex, age, the educational level, the area of residence, kidney disease, or high HbA1c values [47,49,56]. These differences could be due to the difference in the methodologies used and climatic, socio-economic, and cultural factors in each country. Another possible factor is the year in which the study was conducted, as the number of people with diabetes is increasing faster than would be expected from previous studies [2].

There was also a statistically significant association between polymedicated patients and the presence of ONM (*p* = 0.017; OR = 1.974; 95% CI 1.647–2.434). Of the 54 patients with a positive microbiological culture yielding ONM-causing pathogens, 33 were being treated with statins, and of these, 26 had non-dermatophyte fungi or yeasts. This poses a therapeutic challenge as there is a contraindication between statins and itraconazole, one of the most commonly used systemic antifungal treatments. This suggests that laboratory diagnosis should also be performed rather than clinical diagnosis alone, as drugs that are ineffective or even damaging could be prescribed to polymedicated patients and could create resistance.

The high prevalence of ONM among patients with diabetes and diabetic foot is still underestimated [53]. The results of this study suggest that sampling for laboratory testing should be conducted to diagnose and classify fungal infections among patients with diabetic foot. This would allow appropriate treatments to be proposed according to the fungal species and the prevention of aggravation with lesions, ulcers, and amputations.

One of this study’s strengths is that it is the first cross-sectional cohort study to include a population with diabetes and one without diabetes among patients who do not necessarily have clinical signs of nail infection. Furthermore, patients with diabetic foot and a history of ulcers and amputations were included, and the diagnosis of ONM was made by combining two laboratory tests, thus reducing false negatives. Propensity scoring was also used to avoid the non-randomisation bias characteristic of observational studies, and the sample was considerably large and representative.

However, this study also has some limitations that should be noted. The study involved only one centre, and the results may differ between centres. Furthermore, all patient assessments and culture sampling were performed in a podiatric clinic specialising in ONM and diabetic foot. Thus, the results may differ from those in dermatology centres or podiatry practises not specialising in this type of patient.

In conclusion, the results of this study suggest that the early diagnosis of ONM and knowledge of risk factors among patients with diabetes could help to prevent ONM through the education of professionals and patients. This could help to prevent complications among patients with diabetes and avoid serious injuries among patients with diabetic foot. Future research should include the early detection of these risk factors and the influence of health education in reducing the prevalence of such infections and their complications.

## Figures and Tables

**Table 1:** Characteristics, comorbidities, and risk factors of the study population.

	Total (N = 320) n (%)	Diabetes (N = 160) n (%)	No Diabetes (N = 160) n (%)
Sex			
Men	184 (57.5)	128 (80)	56 (35)
Women	136 (42.5)	32 (20)	104(65)
BMI range:			
Underweight (<18.5%)	5 (1.6)	0 (0)	5 (3.1)
Normal (18.5–24.9)	107 (33.4)	52 (32.5)	55 (34.4)
Overweight (24.9–29.9)	122 (38.1)	62 (38.8)	60 (37.5)
Obesity (>29.9)	86 (26.9)	46 (28.7)	40 (25)
Infectious diseases	8 (2.5)	4 (2.5)	4 (2.5)
Sedentary lifestyle	252 (78.8)	148 (92.5)	104 (65)
Smoking	48 (15)	20 (12.5)	28 (17.5)
Alcohol	15 (4.7)	12 (7.5)	3 (1.9)
Occlusive footwear	7 (2.2)	2 (1.3)	5 (3.1)
Occupation			
Online work	11 (3.4)	5 (3.1)	6 (3.8)
Administrative	15 (4.7)	6 (3.8)	9 (5.6)
Requires activity	52 (16.3)	14 (8.8)	38 (23.8)
Unemployed	20 (6.3)	13 (8.1)	7 (4.4)
Retired	222 (69.4)	122 (76.3)	100 (62.5)
History of ulcers	126 (39.4)	118 (73.8)	8 (5)
History of minor amputations	48 (15)	45 (28.1)	3 (1.9)
Revascularisations	26 (8.1)	20 (12.5)	6 (3.8)
HT	162 (50.6)	112 (70)	50 (31.3)
Cholesterol	180 (56.3)	110 (68.8)	70 (43.3)
Neuropathy	110 (34.4)	105 (65.6)	5 (3.1)
Nephropathy	50 (15.6)	37 (23.1)	13 (8.1)
Retinopathy	94 (29.4)	77 (48.1)	17 (10.6)
Cardiovascular history	90 (28.1)	64 (40)	26 (16.3)
Endocrine control	102 (31.9)	95 (59.4)	7 (4.4)
Arthritis	45 (14.1)	23 (14.4)	22 (13.8)
Arthrosis	112 (35)	43 (26.9)	69 (43.1)
PVD	108 (33.8)	78 (48.8)	30 (18.8)
Arterial calcification	29 (9.1)	28 (17.5)	1 (0.6)
Anti-aggregants	116 (36.3)	94 (58.8)	22 (13.8)
OAC	62 (19.4)	47 (29.4)	15 (9.4)
Cholesterol medication	168 (52.5)	109 (68.1)	59 (36.9)
HT medication	156 (48.8)	110 (68.8)	46 (28.7)
Polymedicated	138 (43.1)	123 (76.9)	15 (9.4)
Area of residence			
City	267 (83.4)	128 (80)	139 (86.9)
Rural	53 (16.6)	32 (20)	21 (13.1)
Level of education			
Primary	62 (19.4)	39 (24.4)	23 (14.4)
Secondary	33 (10.3)	18 (11.3)	15 (9.4)
Bachelor’s degree	115 (35.9)	68 (42.5)	47 (29.4)
University	110 (34.4)	35 (21.9)	75 (46.9)
Active ulcers	49 (15.3)	45 (28.1)	4(2.5)
Diabetes type			
DM1	-	23 (14.4)	-
DM2	-	137 (85.6)	-
Antidiabetics			
OAD	-	70 (43.8)	-
Insulin	-	37 (23.1)	-
OAD + insulin	-	53 (33.1)	-
Diabetic foot	-	131 (81.9)	-
Hba1C ranges			
0–7%	-	96 (60)	-
>7%	-	64 (40)	-
Blood glucose			
<126 mg/dL	-	72 (45)	-
>126 mg/dL	-	88 (55)	-

BMI: body mass index; HT: hypertension; PVD: peripheral vascular disease; OAC: anticoagulant; HbA1c: glycosylated haemoglobin; DM1: type 1 diabetes mellitus; DM2: type 2 diabetes mellitus; OAD: oral antidiabetic.

**Table 2:** Characteristics, comorbidities, and risk factors of the study sample expressed as the mean, standard deviation, minimum, and maximum values of each variable.

	Total N = 320	Diabetes N = 160	No Diabetes N = 160
Age (years)	66.92 ± 11.87 [18–96]	68.41 ± 10.85 [45–96]	65,44 ± 12.67 [18–95]
Weight (Kg)	78.31 ± 16.58 [43–170]	81.98 ± 17.08 [46–170]	74.65 ± 15.26 [43–129]
BMI	27.61 ± 12.40 [14.45–35.29]	28.57 ± 16.94 [19.03–35.29]	26.65 ± 4.46 [14.45–37.37]
Total medication	4.77 ± 4.071 [0–26]	7.34 ± 3.99 [1–26]	2.21 ± 2.01 [0–10]
Number of affected nails	n = 87 2.22 ± 2.04 [1–10]	n = 59 2.19 ± 2.07 [1–10]	n = 28 2.32 ± 2.00 [1–10]
OSI score (0–35)	n = 87 22.08 ± 7.63 [2–35]	n = 59 21.85 ± 7.77 [4–35]	n = 28 22.57 ± 7.46 [2–35]
Basal blood glucose (mg/dL)	-	135,18 ± 38.84 [61–390]	-
HbA1C (%)	-	7.09 ± 1.32 [4.5–12.10]	-
Evolution diabetes (years)	-	23.74 ± 14.11 [1–68]	-

OSI: onychomycosis severity index; HbA1c: glycosylated haemoglobin.

**Table 3:** Characteristics of the sample with clinical signs of infection and with the presence of onychomycosis after diagnosis by combined microbiological culture and PCR.

	Total n (%)	Diabetes n (%)	No Diabetes n (%)
Ulcers under the nail plate	n = 320 5 (1.6)	n = 160 5 (3.1)	n = 160 0 (0)
Clinical signs of ONM	n = 320 125 (39.1)	n = 160 86 (53.8)	n = 160 39 (24.4)
Nail thickening	n = 125 90 (72)	n = 86 61 (70.9)	n = 39 29 (74.4)
Subungual hyperkeratosis	n = 125 88 (70.4)	n = 86 62 (72.1)	n = 39 26 (66.7)
Chromonychia	n = 125 93 (74.4)	n = 86 63 (73.3)	n = 39 30 (76.9)
Onycholysis	n = 125 31 (24.8)	n = 86 11 (6.9)	n = 39 20 (51.3)
Dermatophytoma	n = 125 11 (8.8)	n = 86 6 (7)	n = 39 5 (12.8)
Detritus	n = 125 42 (33.9)	n = 86 32 (37.6)	n = 39 10 (25.6)
Fragile nail	n = 125 13 (10.5)	n = 86 12 (14.1)	n = 39 1 (2.6)
Longitudinal striae	n = 125 17 (13.8)	n = 86 14 (16.5)	n = 39 3 (7.9)
Presence of ONM (PCR + culture)	n = 320 87 (27.19)	n = 160 59 (36.88)	n = 160 28 (17.5)
Microorganism detected by culture	n = 54	n = 42	n = 12
*T. rubrum*	11 (20.4)	9 (21.4)	2 (16.7)
Mixed *	15 (27.8)	13 (31)	2 (16.7)
*Candida* sp.	14 (26)	12 (28.6)	2 (16.7)
*T. mentagrophytes*	2 (3.7)	2 (4.8)	0 (0)
*Aspergillus niger*	2 (3.7)	0 (0)	2 (16.7)
*Penicillium* sp	4 (7.4)	3 (7.1)	1 (8.3)
*Curvularia* sp.	1 (1.9)	1 (2.4)	0 (0)
*Cladosporium* sp.	3 (5.6)	2 (4.8)	1 (8.3)
*T. violacium*	1 (1.9)	0 (0)	1 (8.3)
*A. flavus*	1 (1.9)	0 (0)	1 (8.3)
Type of fungi	n = 87	n = 59	n = 28
Dermatophyte	47 (54)	28 (47.5)	19 (67.9)
Mould	11 (12.6)	6 (10.2)	5 (17.9)
Yeast	14 (16.1)	12 (20.3)	2 (7.1)
Mixed	15 (17.2)	13 (22)	2 (7.1)
Type of ONM	n = 87	n = 59	n = 28
Distal	9 (10.3)	4 (6.8)	5 (17.9)
Distal–lateral	18 (20.7)	16 (27.1)	2 (7.1)
Superficial	11 (12.6)	8 (13.6)	3 (10.7)
Dystrophic	49 (56.3)	31 (52.5)	18 (64.3)
OSI evaluation	n = 87	n = 59	n = 28
Mild	2 (2.3)	1 (1.7)	1 (3.6)
Moderate	14 (16.1)	10 (16.9)	4 (14.3)
Severe	71 (81.6)	48 (81.4)	23 (82.1)
Area affected	n = 87	n = 59	n = 28
1–10%	1 (1.1)	1 (1.7)	0 (0)
11–25%	19 (21.8)	13 (22)	6 (21.4)
25–50%	21 (24.1)	16 (27.1)	5 (17.9)
51–76%	25 (28.7)	18 (30.5)	7 (25)
>76%	21 (24.1)	11 (18.6)	10 (35.7)
Nail quarter affected	n = 87	n = 59	n = 28
Distal	2 (2.3)	0 (0)	2 (7.1)
Distal not exceeding midline	15 (17.2)	11 (18.6)	4 (14.3)
Exceeding midline	16 (18.4)	15 (25.4)	1 (3.6)
Entire nail without lunula	34 (39.1)	22 (37.3)	12 (42.9)
Involvement of the lunula	20 (23)	11 (18.6)	9 (32.1)
Presence of dermatophytoma	n = 87 10 (11.5)	n = 59 7 (11.9)	n = 28 3 (10.7)
Presence of subungual hyperkeratosis	n = 87 69 (79.3)	n = 59 49 (83.1)	n = 28 20 (71.4)

Mixed infections *: *Candida* sp. + dermatophyte (10/15); *Candida* sp. + Trichophyton rubrum (1/15); *Candida* sp. + *Fusarium* sp. (1/15); *Candida albicans* + *Trichophyton rubrum* (1/15); *Candida* sp. + *Fusarium* sp. (1/15); *Aspergillus* sp. + dermatophyte (1/15). Cases where the dermatophyte pathogen is not specified were identified by PCR but not by microbiological culture. ONM: onychomycosis; OSI: Onychomycosis Severity Index.

**Table 4:** Association between comorbidities and the presence of onychomycosis in the whole population, the population with diabetes, and the population without diabetes. * Variables with *p* < 0.2 were included in the multivariate analysis (Table 5). HT: hypertension; OAC: anticoagulants; PVD: peripheral vascular disease; DM: diabetes mellitus; HbA1c: glycosylated haemoglobin. NA: not applicable.

	Total		Diabetes		No Diabetes	
	*p*-Value	OR [95% CI]	*p*-Value	OR [95% CI]	*p*-Value	OR [95% CI]
Sex	0.012 *	**1.953 [1.159–3.292]**	0.935	1.034 [0.464–2.303]	0.166 *	**0.557 [0.244–1.275]**
Age	0.903	1.001 [0.981–1.022]	0.565	1.009 [0.979–1.039]	0.236	0.981 [0.952–1.012]
Obesity	0.896	0.999 [0.978–1.020]	0.860	0.998 [0.978–1.019]	0.256	0.947 [0.862–1.040]
Sports	0.110 *	**1.171 [0.885–3.334]**	0.497	0.623 [0.159–2.445]	0.930	1.039 [0.443–4.435]
Smoker	0.472	1.304 [0.633–2.688]	0.497	0.704 [0.255–1.942]	0.956	1.030 [0.355–2.993]
Closed footwear	0.356	2.045 [0.448–9.237]	0.702	1.724 [0.106–28.088]	0.202	3.308 [0.526–20.788]
History of ulcers	0.481	1.197 [0.726–1.975]	0.017 *	**2.408 [1.173–4.946]**	0.999	NA
History of amputations	0.158 *	**1.742 [0.806–3.767]**	0.007 *	**3.007 [1.358–6.974]**	0.999	NA
History of revascularisation	0.345	1.625 [0.593–4.452]	0.104 *	**2.588 [0.822–8.149]**	0.956	0.941 [0.119–9.468]
HT	0.135 *	**0.685 [0.417–1.126]**	0.802	1.094 [0.541–2.213]	0.737	0.857 [0.349–2.104]
Cholesterol	0.201	0.720 [0.435–1.191]	0.119 *	**1.780 [0.862–3.676]**	0.177 *	**0.551 [0.232–1.308]**
Neuropathy	0.108 *	**1.517 [0.913–2.521]**	0.349	0.726 [0.371–1.419]	0.999	NA
Nephropathy	0.130 *	**1.639 [0.865–3.104]**	0.194 *	**1.639 [0.777–3.457]**	0.350	0.370 [0.046–2.971]
Retinopathy	0.343	1.292 [0.761–2.195]	0.264	0.693 [0.363–1.324]	0.491	1.526 [0.458–5.083]
Cardiovascular history	0.069 *	**0.613 [0.361–1.039]**	0.014 *	**2.281 [1.181–4.409]**	0.999	NA
Endocrine control	0.051 *	**1.668 [0.997–2.788]**	0.313	0.715 [0.373–1.371]	0.090 *	**3.840 [0.809–18.221]**
Arthritis	0.319	0.709 [0.361–1.394]	0.105 *	**2.089 [0.857–5.092]**	0.609	0.714 [0.196–2.599]
Arthrosis	0.051 *	**1.720 [0.997–2.966]**	0.493	0.772 [0.369–1.617]	0.200	0.568 [0.240–1.349]
PVD	0.372	1.274 [0.748–2.169]	0.028 *	**2.093 [1.084–4.040]**	0.507	0.679 [0.470–4.611]
Calcification	0.412	1.479 [0.581–3.764]	0.068 *	**0.407 [0.389–1.428]**	1.000	NA
Anti-aggregants	0.520	0.847 [0.510–1.406]	0.376	0.746 [0.389–1.428]	0.276	0.431 [0.095–1.960]
OAC	0.716	0.892 [0.408–1.649]	0.905	0.958 [0.472–1.942]	0.271	0.312 [0.039–2.477]
Cholesterol medication	0.277	0.759 [0.462–1.248]	0.325	1.425 [0.704–2.885]	0.157 *	**0.513 [0.204–1.292]**
HT medication	0.250	0.748 [0.457–1.226]	0.842	0.932 [0.467–1.860]	0.630	0.795 [0.313–2.022]
Number of medications	0.031 *	**1.066 [1.006–1.130]**	0.964	1.002 [0.924–1.086]	0.312	0.888 [0.706–1.118]
Polymedicated	0.008 *	**1.956 [1.188–3.218]**	0.524	1.288 [0.591–2.806]	0.271	0.312 [0.039–2.477]
Rural area	0.004 *	**2.432 [1.319–4.483]**	0.036 *	**2.321 [1.057–5.094]**	0.159 *	**2.127 [0.744–6.083]**
Level of education	0.054 *	**1.764 [0.908–3.427]**	0.064 *	**1.452 [0.506–3.172]**	0.769	1.367 [0.384–3.817]
Current ulcer	0.05 *	**1.891 [1.000–3.575]**	0.381	1.370 [0.677–2.773]	0.999	NA
Profession	0.293	0.427 [0.046–1.590]	0.700	0.214 [0.019–2.477]	0.598	0.361 [0.034–7.452]
Type of DM	-	-	0.251	1.788 [0.663–4.822]	-	-
DM evolution	-	-	0.276	0.987 [0.964–1.011]	-	-
Diabetic foot	-	-	0.163 *	**0.561 [0.249–1.264]**	-	-
HbA1c	-	-	0.033 *	**2.039 [1.057–3.930]**	-	-
Basal blood glucose	-	-	0.032 *	**2.069 [1.064–4.026]**	-	-
Diabetes medication	-	-	0.960	1.010 [0.698–1.460]	-	-
**Clinical signs of ONM infection**						
Thickening	0.148 *	**1.833 [0.806–4.171]**	0.938	1.040 [0.383–2.827]	0.015 *	**7.200 [1.468–35.317]**
Subungual hyperkeratosis	0.005 *	**3.221 [1.426–7.277]**	0.006 *	**4.052 [1.491–11.010]**	0.319	2.083 [0.492–8.815]
Chromonychia	0.571	0.781 [0.332–1.838]	0.523	0.706 [0.243–2.054]	0.047 *	**5.000 [1.019–24.525]**
Onycholysis	0.278	0.593 [0.230–1.526]	0.322	2.250 [0.452–11.207]	0.649	1.385 [0.341–5.615]
Detritus	0.115 *	**2.006 [0.845–4.765]**	0.132 *	**2.165 [0.792–5.916]**	0.507	1.800 [0.317–10.232]
Fragile nail	0.534	0.651 [0.169–2.515]	0.589	1.469 [0.364–5.928]	1.000	NA
Longitudinal striae	0.287	1.773 [0.618–5.089]	0.333	0.569 [0.173–1.813]	0.775	0.692 [0.056–8.581]

**Table 5:** Association between comorbidities and the presence of onychomycosis in the whole population, the population with diabetes, and the population without diabetes according to multivariate analysis including variables *p* < 0.2 from the univariate analysis. * Variables *p* < 0.05 were considered statistically significant. AHT: hypertension; PVD: peripheral vascular disease; HbA1c: glycosylated haemoglobin.

	Total Multivariate		Diabetes Multivariate		No Diabetes Multivariate	
	*p*-Value	OR [95% CI]	*p*-Value	OR [95% CI]	*p*-Value	OR [95% CI]
Sex	0.080	1.774 [0.857–3.369]	-	-	0.256	0.611 [0.261–1.430]
Sports	0.678	1.175 [0.550–2.509]	-	-	-	-
History of ulcers	-	-	0.076	3.474 [0.876–13.771]	-	-
History of amputations	0.002 *	**4.353 [1.728–10.966]**	0.014 *	**4.493 [1.356–14.881]**	-	-
History of revascularisation	-	-	0.04 *	**5.879 [1.083–31.922]**	-	-
HT	0.852	1.064 [0.555–2.042]	-	-	-	-
Cholesterol	-	-	0.369	0.652 [0.257–1.657]	0.819	0.839 [0.186–3.780]
Neuropathy	0.905	1.041 [0.496–1.861]	-	-	-	-
Nephropathy	0.384	1.398 [0.557–1.522]	0.265	0.569 [0.211–1.533]	-	-
Cardiovascular history	0.307	1.401 [0.373–1.463]	<0.001 *	**10.046 [3.322–30.381]**	-	-
Endocrine control	0.460	1.278 [0.408–1.500]	-	-	0.150	3.307 [0.648–16.879]
Arthritis	-	-	0.241	2.242 [0.582–8.643]	-	-
Arthrosis	0.014 *	**2.264 [1.183–4.333]**	-	-	-	-
PVD	-	-	0.074	0.377 [0.130–1.099]	-	-
Calcification	-	-	0.184	0.423 [0.119–1.503]	-	-
Cholesterol medication	-	-	-	-	0.674	0.710 [0.144–3.504]
Number of medications	0.702	0.979 [0.878–1.091]	-	-	-	-
Polymedicated	**0.017**	**1.974 [1.647–2.434]**	-	-	-	-
Rural area	0.015 *	**2.376 [1.185–4.765]**	0.267	1.880 [0.617–5.728]	0.142	2.242 [0.763–6.588]
Level of education	0.014 *	**1.506 [1.407–3.560]**	0.006 *	**1.478 [1.210–4.796]**	-	-
Current ulcer	0.439	1.380 [0.610–3.121]	-	-	-	-
Diabetic foot	-	-	0.672	1.389 [0.304–6.341]	-	-
HbA1c	-	-	0.008 *	**4.036 [1.448–11.253]**	-	-
Basal blood glucose	-	-	0.270	1.713 [0.658–4.458]	-	-
**Clinical signs of ONM infection**						
Thickening	0.043 *	**2.537 [1.029–6.259]**	-	-	0.010 *	**12.135 [1.806–81.518]**
Subungual hyperkeratosis	<0.001 *	**4.495 [1.839–10.984]**	0.001 *	**6.573 [2.060–20.976]**	-	-
Chromonychia	-	-	-	-	0.026 *	**9.232 [1.303–65.388]**
Detritus	0.036 *	**2.766 [1.070–7.149]**	0.032 *	**3.660 [1.117–11.995]**	-	-

## Data Availability

The original contributions presented in the study are included in the article, further inquiries can be directed to the corresponding author.
